# Research on Quantitative Analysis Technology for Pacific White Shrimp Density Based on Environmental DNA

**DOI:** 10.1002/ece3.73299

**Published:** 2026-04-14

**Authors:** Guangyan Liang, Sizhe Wang, Shan Wang, Yusheng Jiang, Yuxue Qin

**Affiliations:** ^1^ College of Marine Science and Environment Dalian Ocean University Dalian China; ^2^ Dalian Key Laboratory of Shrimp and Crab Breeding and Healthy Aquaculture Dalian Ocean University Dalian China

**Keywords:** aquaculture monitoring, biomass quantification, environmental DNA, *Litopenaeus vannamei*

## Abstract

*Litopenaeus vannamei* is one of the most essential farmed shrimp species globally, yet disease outbreaks during cultivation continue to hinder industry development. Accurate biomass estimation is critical for optimizing feeding, maintaining water quality, and preventing disease. This study developed a quantitative method for environmental DNA (eDNA) using real‐time fluorescence quantitative PCR with *L. vannamei* as the model species. Species‐specific primers were designed and validated to ensure detection specificity. Temperature significantly influenced eDNA degradation, with higher temperatures accelerating decay; eDNA remained detectable in water for over a month at 26°C. A strong linear correlation was observed between eDNA concentration and shrimp biomass, highlighting eDNA's potential for biomass assessment. eDNA release per unit body weight varied with growth stage, following the pattern “small > medium > large,” while short‐term feeding had no significant effect. The method was validated in both indoor recirculating systems and outdoor ponds, where eDNA levels correlated positively with final yield. This study establishes a robust eDNA‐based framework for monitoring shrimp biomass, enabling precise, dynamic management in aquaculture.

## Graphical abstract

This study developed an environmental DNA (eDNA)‐based quantitative method using real‐time fluorescence quantitative PCR to estimate the biomass of *Litopenaeus vannamei* , a key farmed shrimp species. It found temperature accelerates eDNA degradation (though eDNA remains detectable for over a month at 26°C), a strong linear correlation between eDNA concentration and shrimp biomass, and that eDNA release per unit weight follows “small > medium > large” across growth stages, with validation in indoor and outdoor systems confirming eDNA's potential for precise aquaculture biomass monitoring.

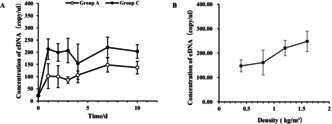

## 1 Introduction

*Litopenaeus vannamei* is among the most prominent shrimp species in global aquaculture, valued for its high protein and low fat content, palatable flavor, and strong consumer demand (Li et al. 2021). The species sustains stable market demand and substantial economic returns in both domestic and international markets, driving the continuous expansion of farming operations. According to 2024 statistics, *L. vannamei* accounts for approximately 83% of global farmed shrimp production, generating over 4 billion US dollars in annual economic value, establishing it as a cornerstone of the global shrimp industry (FAO 2020, 2023). Despite its economic significance, disease outbreaks remain a significant constraint on sustainable development. Inaccurate estimates of total biomass in culture ponds often lead to overfeeding, resulting in the accumulation of residual feed at the pond bottom. The accumulation of organic matter acts as a “fuse” for the deterioration of water quality. It does so by disrupting the oxygen supply, releasing toxins, altering the chemical environment, and fostering the growth of pathogenic organisms (Gyamfi et al. 2022; Heri Ariadi et al. 2025). This organic buildup degrades water quality and increases the risk of pathogen proliferation (Ridlo et al. 2024). Poor biomass assessment is closely associated with the incidence of prevalent diseases such as white spot syndrome, yellow head disease, and mononuclear cell proliferation (Mai and Dhar 2025; Nuanpirom et al. 2025; Yaemkasem et al. 2022). These diseases not only increase larval mortality but also impair growth performance and reduce marketable yield, resulting in significant economic losses in shrimp aquaculture (Musa et al. 2022). Therefore, accurate biomass assessment is critical for enabling precise feeding strategies, mitigating disease risks, and enhancing overall farming efficiency.

However, traditional approaches for estimating prawn biomass are predominantly based on trawling and mark‐recapture techniques. Trawling is a labor‐ and resource‐intensive sampling method that is inherently prone to substantial bias. Particularly due to gear selectivity, habitat avoidance by target species, and inconsistent capture efficiency across heterogeneous pond environments. Thereby compromising its reliability for accurately characterizing the spatial distribution and temporal dynamics of prawn populations in aquaculture ponds (Rijnsdorp et al. 1997; Santucci et al. 2024; Yang et al. 2018). The mark‐recapture method is an ecological statistical technique that involves capturing, marking, releasing, and recapturing individuals to estimate population abundance from recapture rates (Casas and Saborido‐Rey 2023). It is operationally complicated. The marking process can cause physical damage to individuals, potentially compromising their growth and survival. Moreover, it is not applicable to juvenile prawns due to their small body size and physiological fragility (Kneib and Huggler 2001; Marvin et al. 2015). In addition, both methods are invasive, which prevents frequent or continuous monitoring (Moreira‐Ferreira et al. 2020). With the expansion and intensification of aquaculture, the technical limitations of conventional methods have become increasingly prominent. Therefore, there is an urgent demand for efficient, accurate, and non‐invasive techniques for biomass assessment. Environmental DNA (eDNA) technology can effectively overcome these shortcomings. Through high‐throughput sequencing (HTS), eDNA enables rapid detection of extensive species‐specific information with sensitivity far superior to traditional fishing or mark‐recapture approaches, while being entirely non‐invasive.

Environmental DNA (eDNA) is defined as genetic material obtained directly from environmental samples (e.g., water, sediment, soil) without the need for visual observation or capture of the target organisms (Thomsen and Willerslev 2015). As a non‐invasive molecular tool, eDNA has been widely adopted for biodiversity monitoring and species identification in aquatic ecosystems. In particular, it has been successfully applied to assess fish and mollusk communities, demonstrating strong potential for rapid, efficient monitoring in aquaculture environments. Beyond species composition, eDNA also enables sensitive detection of aquatic pathogens, such as White Spot Syndrome Virus (WSSV), by targeting specific genetic fragments released into the surrounding environment (Li et al. 2025). This means that eDNA can not only characterize biological communities in water bodies but also effectively track bacterial abundance and pathogen loads in aquaculture systems, providing timely data to support health management and disease prevention. Compared with conventional survey methods, eDNA offers clear advantages, including lower labor input, reduced ecological disturbance, and rapid, high‐sensitivity detection (Schmidt et al. 2021). Nevertheless, the reliability of eDNA signals can be affected by various environmental factors. Temperature, pH, salinity, and water chemistry can influence eDNA persistence and degradation, thereby affecting detection sensitivity and causing variability across different environmental conditions (Çevik and Çevik 2025). Despite these constraints, eDNA‐based quantification has advanced considerably in recent years. For instance, robust quantitative models describing eDNA shedding and degradation dynamics have been well established for various marine invertebrates and fish species (Chouhan et al. 2023; Kwong et al. 2021; Peters et al. 2018; Sassoubre et al. 2016).

In recent years, eDNA has proven effective for species identification and biodiversity assessment in fish and mollusk populations, highlighting its potential for rapid monitoring in aquaculture settings. For crustaceans, recent studies have confirmed that *L. vannamei* releases detectable eDNA into culture ponds, with concentrations reaching up to 10^6^ copies L^−1^, and these levels are significantly and positively correlated with actual shrimp biomass (Li et al. 2022). However, several critical limitations hinder accurate and consistent eDNA‐based biomass estimation in practical aquaculture. First, highly species‐specific shrimp primers remain limited, and most available primers target conserved crustacean sequences, which increases the risk of non‐specific cross‐amplification and reduces quantification accuracy (Wang et al. 2021). Second, environmental parameters strongly affect eDNA degradation: eDNA copy numbers gradually decrease after removal of the source organism (Eichmiller et al. 2016; McCartin et al. 2022), and vertical distribution patterns show higher eDNA concentrations in bottom waters than in surface layers, influenced by shrimp behavior and limited water mixing (Fukumori et al. 2024; McCartin et al. 2025). Third, eDNA shedding rates vary ontogenetically: differences in metabolic activity lead to lower eDNA release in juvenile fish, which produce approximately 60% of the eDNA detected in adults (Maruyama et al. 2014). Currently, comprehensive quantitative models that integrate biological (e.g., ontogenetic variation) and environmental (e.g., degradation, distribution) drivers remain underdeveloped. Therefore, to achieve reliable and accurate biomass assessment for *L. vannamei* , key technical challenges must be addressed, including improving primer specificity, accounting for environmental effects on eDNA degradation, and characterizing life‐stage‐dependent eDNA shedding. Against this background, the present study aims to develop a novel eDNA‐based framework for evaluating *L. vannamei* populations, with the ultimate goal of providing a robust scientific basis for optimizing monitoring and management strategies in commercial shrimp aquaculture.

## 2 Materials and Methods

### 2.1 Culture Treatment of *L. vannamei*

The prawns used in this experiment were sourced from Panjin Guanghe Crab Industry Co. Ltd., where only *L. vannamei* is cultured. Selected individuals were held in white tanks for 2 days without feeding. Before stocking the shrimp, three repeated eDNA samplings were conducted in the reservoir water after it was filled, serving as control samples to evaluate the potential presence of residual eDNA from the target species in the water.

### 2.2 Design of *L. vannamei* Mitochondrial COI Gene‐Specific Primers

Specific primers targeting the mitochondrial cytochrome c oxidase subunit I (COI) gene of *L.vannamei* were designed. The COI gene sequence of *L. vannamei* was retrieved from the NCBI GenBank database (https://www.ncbi.nlm.nih.gov/). Homologous COI sequences from closely related and other co‐occurring crustacean species were also downloaded for multiple sequence alignment. Sequence alignment was conducted using BioEdit and MEGA7 software. Based on the alignment results, a region with high interspecific sequence divergence was selected to ensure species specificity. Primers were designed de novo using Primer Premier 6 and Beacon Designer 8 software. The primer sequences were as follows: Nanmeibai‐F (5′–3′): TGGCGTCCGCTTCGCAGTCT; Nanmeibai‐R (5′–3′): CCCAGAACATCTAAGGGCATCACA. Primers were commercially synthesized by Sangon Biotech (Shanghai) Co. Ltd. Specificity was verified by PCR amplification and agarose gel electrophoresis using genomic DNA extracted from muscle tissues of the crustacean species that are the only cultured species present at the aquaculture farm (Table 1). This validation was performed to confirm the specificity of the primer pair under actual field detection conditions and to ensure strict species‐specific amplification.

**TABLE 1:** Sample‐specific information.

DNA sample ID	Species name	Source of the sample
Fan	*Litopenaeus Vannamei*	Muscle tissue
He	*Eriocheir sinensis*	Muscle tissue
Zhong	*Fenneropenaeus chinensis*	Muscle tissue
Kou	*Oratosquilla oratoria*	Muscle tissue
Meng	*Moina mongolica*	Muscle tissue

*Note:* Fan: *Litopenaeus vannamei* ; He: *Eriocheir sinensis* ; Zhong: *Fenneropenaeus chinensis* ; Kou: *Oratosquilla oratoria*; Meng: *Moina mongolica* . The five species listed above are the most common crustaceans on this farm. Therefore, four other crustaceans were deliberately selected for comparison with *L. vannamei* .

### 2.3 eDNA Degradation Experiments of *L. vannamei* at Different Temperatures

#### 2.3.1 Experimental Treatment and Sample Collection

The experiment was conducted in 100‐l plastic tanks with dimensions of 50.0 cm × 36.5 cm × 62.5 cm. Each tank was filled with 50 L of culture water, maintaining a salinity of 24‰ and a temperature range of 28.8°C–30.5°C. At the end of the acclimation period, the biomass of *L. vannamei* in the pond was approximately 75 kg, with an average individual weight of 8.3 g. A water sample and several shrimp were collected from the pond and transferred to a pre‐sterilized white plastic bucket. After adding fresh culture water, these organisms were reared under controlled conditions for another 10 days. Finally, 1 L of surface water was collected from each replicate tank in all groups. Three temperature treatment groups were set up: Group A (2°C, refrigerated in a refrigerator), Group B (14°C, cultured in a constant‐temperature incubator), and Group C (26°C, room temperature), with three replicates per group. Corresponding control samples without shrimp were also prepared under the same three temperature conditions to monitor background eDNA and potential contamination. Shrimp were removed from the water at the beginning of the experiment (time 0 h), and all subsequent samples were collected from the water without shrimp. Water samples were collected from each treatment and control group at designated time points: 0 h, 3 h, 6 h, 12 h, 1 days, 2 days, 4 days, and 8 days. To investigate the long‐term persistence characteristics of eDNA in the water body, additional sampling was conducted for Group C at 12 days, 14 days, 16 days, 19 days, 22 days, and 28 days. Sampling bottles were fitted with a 400 mesh nylon net (about 40 μm) over the opening to prevent large particulate matter from entering for each replicate at each sampling time. All samples were immediately sealed in black plastic bags to minimize light exposure and stored under the corresponding temperature conditions.

#### 2.3.2 Determination Test of eDNA Degradation Rate

The eDNA degradation kinetic curve of each temperature group was fitted with culture time as the independent variable and eDNA concentration as the dependent variable, and the eDNA degradation rate per unit time was calculated. The degradation rate formula of eDNA is shown as (1).

(1) $$\ln\;\left(C_{t}\right)=-\mathrm{kt}+\ln\;\left(C_{0}\right)$$

Here, *k* is the degradation rate, *t* represents time, *C*_0_ is the eDNA concentration at *t* = 0, and *C*_*t*_ is the eDNA concentration at time t.

### 2.4 eDNA‐Based Quantification of *L.Vannamei* Biomass

This experiment was also conducted in a 100‐l plastic water tank with dimensions of 50.0 cm × 36.5 cm × 62.5 cm. Each tank was filled with 50 L of culture water, maintained at a salinity of 24‰ and a temperature of 28.8°C–30.5°C. No water exchange was performed during the experimental period, and all *L.vannamei* were placed on a fasting regimen. The average weight of the adult prawns used in the experiment was 21.3 ± 1.24 g. Four biomass treatment groups (A1, A2, A3, and A4) were established, each with three replicate tanks. Shrimp densities were 1, 2, 3, and 4 individuals per tank, yielding mean biomass of 23.1 ± 1.39 g, 40.67 ± 2.57 g, 62.3 ± 5.37 g, and 83.7 ± 1.33 g per tank, respectively. Surface water samples (1 L) were collected from each replicate tank of groups A1 and A3 on Days 1, 2, 3, 4, 7, and 10 after shrimp introduction. Additionally, on Day 7, 1 L of surface water was collected from all replicate tanks within each of the four groups. The collected water samples were immediately filtered (common filtration methods are described in Section 2.6 below). Meanwhile, source water samples were collected to determine the baseline eDNA concentration in the culture water supply.

### 2.5 Experiments on Different Growth Stages of *L. vannamei*

The experiment was divided into two parts. In the first part, two experimental groups were set up: the feeding group and the fasting group. Each group contained three adult shrimp of the same size, and each group had three replicate tanks. During the experiment, the shrimp in the feeding group were fed four times a day at regular intervals, while the shrimp in the fasting group were not fed. Uneaten feed and fecal matter generated during cultivation were removed from the feeding group tanks three times daily (morning, midday, and evening) using sterilized tools to maintain water quality. No water exchange was performed in any of the tanks throughout the experimental period. In the second part, three treatment groups (L, M, and S) were established, each with three replicates. The L group contained three shrimp per tank with an average individual weight of 19.23 ± 0.31 g (total biomass ≈60 g). The M group contained multiple shrimp per tank with an average individual weight of 3.5 ± 0.36 g (total biomass ≈36 g), and the S group contained multiple shrimp per tank with an average individual weight of 0.3 ± 0.03 g (total biomass ≈22 g). All groups in this phase were maintained under fasting conditions, and no water replacement was conducted. On Day 6 post‐stocking, 1 L of surface water was collected from each replicate tank across all groups using pre‐labeled sampling bottles. The collected water samples were immediately filtered (common filtration methods are described in Section 2.6 below).

### 2.6 Water Sample Filtration and eDNA Preservation

Samples were filtered using a vacuum filtration system (SIBATA, model WJ‐20) equipped with Whatman glass fiber filters (47 mm diameter, 0.45 μm pore size). Prior to each filtration, the funnel should be bleached and then rinsed with nuclease‐free water to prevent cross‐contamination. After filtration, each filter was fixed by adding a small volume of ethanol for 60 s, and the ethanol was then removed by brief re‐filtration. The filter membranes were then carefully transferred using sterile forceps onto clean filter paper, wrapped in aluminum foil to protect them from moisture and light, and stored at −80°C until eDNA extraction.

### 2.7 eDNA Extraction and Fluorescence Quantitative PCR Analysis

DNA was extracted using the DNeasy Blood and Tissue Kit (Qiagen, Germany) according to the protocol described by Roose‐Amsaleg et al., with minor modifications (Roose‐Amsaleg et al. 2001).

Quantitative analysis of the extracted eDNA samples was performed using the AceQ qPCR SYBR Green Master Mix (Vazyme) according to the manufacturer's instructions. The 20 μL reaction system contained 10 μL of 2 × SYBR Green Fast qPCR Master Mix, 0.5 μL of forward primer (10 μmol/L), 0.5 μL of reverse primer (10 μmol/L), 2 μL of template DNA, and 7 μL of nuclease‐free water. Amplification was conducted using a two‐step cycling protocol: initial denaturation at 95°C for 5 min, followed by 40 cycles of 95°C for 15 s and 60°C for 30 s. Each standard sample and eDNA sample was run in triplicate, and three no‐template controls (NTCs) were included per 96‐well plate to monitor potential contamination. Data were analyzed via absolute quantification. qPCR was carried out on an MA‐6000 Real‐Time PCR System (Suzhou Yarui Biotechnology Co. Ltd.) using Axygen 96‐well plates.

### 2.8 Validation of the Effectiveness of eDNA Detection Technology

To validate the applicability of the eDNA method developed in controlled indoor experiments to field‐based aquaculture systems, surface water samples were collected in the summer of 2021 from three outdoor *L. vannamei* ponds operated by Panjin Guanghe Crab Industry Co. Ltd. (Panjin City, Liaoning Province, China). eDNA sampling was conducted at 4 weeks post‐stocking, which represents the early‐middle culture stage with relatively high shrimp density and low environmental interference (e.g., residual feed and fecal accumulation), enabling the eDNA signal to stably reflect the actual shrimp population in ponds. From each pond, 3 L of surface water was collected and divided into 3 equal aliquots (1 L each), which were immediately filtered on‐site. A capture survey using encircling nets was performed at 8 weeks post‐stocking (the late culture stage prior to harvest), when shrimp had reached marketable size and could be reliably captured for biomass estimation; the total wet weight of the catch from each pond was recorded individually as a proxy for actual biomass.

### 2.9 Data Processing and Statistical Analysis

Data processing and statistical analysis were performed using SPSS 26 and GraphPad Prism 9. One‐way analysis of variance (ANOVA) was applied to assess differences in measured indicators across experimental groups. Post hoc pairwise comparisons were conducted using the least significant difference (LSD) test. Differences were considered statistically significant at *p* < 0.05.

## 3 Results and Analysis

### 3.1 Primer Specificity Verification

**FIGURE 1:**
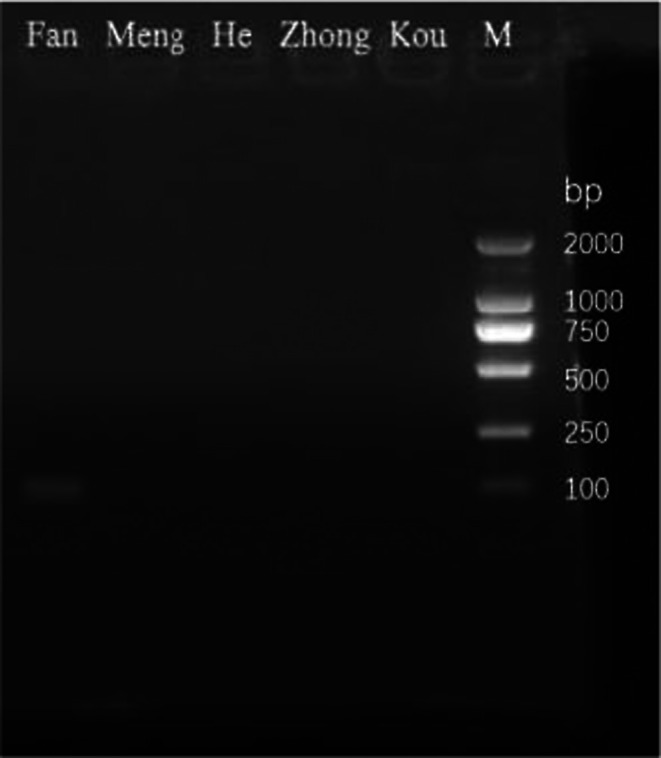
Specific primer verification. M: DNA Marker DL 2000; Lane (From left to right):Fan: *Litopenaeus Vannamei* ；Meng: *Moina mongolica* ；He: *Eriocheir sinensis* ；Zhong: *Fenneropenaeus chinensis* ；Kou: *Oratosquilla oratoria*.

In the present study, the designed primers only produced specific bands for the DNA template of the target species, while no non‐specific amplification bands were observed in the common crustaceans used as controls. No bands were detected in the other control species, further confirming the specificity of the primers in practical detection. Our results indicate that the primer pair Nanmeibai‐F/Nanmeibai‐R amplified a single 112 bp fragment from the control water samples collected prior to shrimp introduction, with no visible amplification detected in the remaining lanes (Figure 1). These results confirm the high specificity of the designed primers for the target species.

### 3.2 eDNA Degradation Model Fitting and Rate Estimation Across Temperatures

**FIGURE 2:**
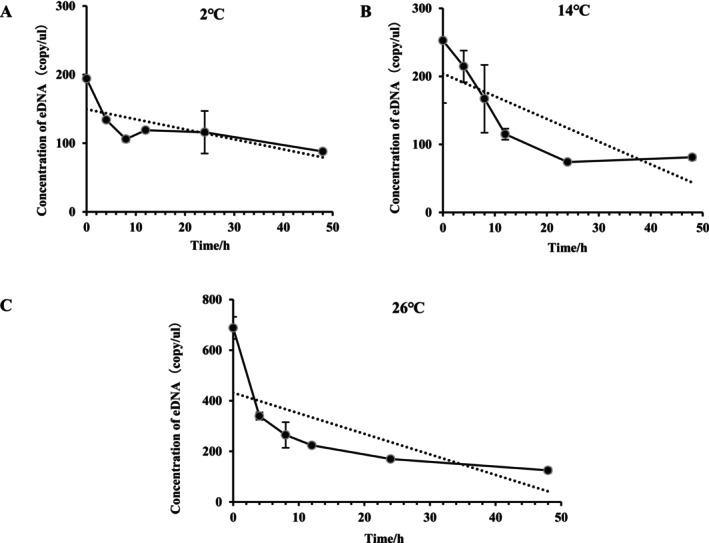
eDNA Dynamics of *L.vannamei* over time at different temperatures.

The concentration of eDNA from *L. vannamei* gradually declined over time across three temperature conditions (Figure 2A–C). Within 8 h, over 50% of the environmental DNA (eDNA) was degraded; thereafter, the degradation rate progressively declined with increasing incubation time (Table 2). The influence of water temperature on eDNA degradation kinetics was further assessed. The experimental results indicated that under the test conditions (2°C, 14°C and 26°C), the eDNA degradation rate decreased significantly over time (*p* < 0.05), showing a clear time‐dependent trend. At different temperatures, the degradation rate increased with the rise in temperature, with the highest rate observed at 26°C. This suggests that temperature significantly affects the degradation rate, but due to the limited sample size, the main effect of temperature did not reach statistical significance.

**TABLE 2:** Degradation rates of eDNA at different temperatures over different time periods.

Temperature (°C)	4 h	8 h	12 h	24 h	48 h
2	1.02 ± 0.00^a^	0.56 ± 0.00^b^	0.36 ± 0.00^bc^	0.18 ± 0.02^cd^	0.10 ± 0.00^d^
14	1.06 ± 0.12^a^	0.54 ± 0.07^b^	0.39 ± 0.07^bc^	0.21 ± 0.03^cd^	0.10 ± 0.01^d^
26	1.46 ± 0.03^a^	0.75 ± 0.03^b^	0.51 ± 0.01^bc^	0.26 ± 0.00^cd^	0.13 ± 0.00^d^

*Note:* Degradation rate (%) data are presented as mean ± standard deviation. Different letters within the same row indicate significant differences (*p* < 0.05). Among them, “a” represents the highest significance, “b” represents relatively high significance, “c” represents relatively low significance, and “d” represents the lowest significance.

### 3.3 eDNA Retention in Water at Room Temperature

**FIGURE 3:**
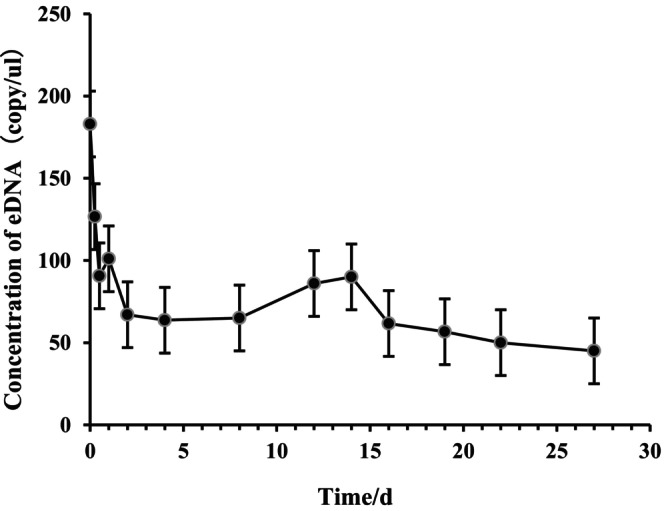
Temporal dynamics of eDNA from *L.vannamei* at 26°C.

Following the removal of *L. vannamei* and the cessation of eDNA release, eDNA concentration in the water declined with time. On Day 1, the DNA copy number was 180 copies per microliter of water sample, decreasing to 44 copies per microliter by Day 26. Within the first 12 h, eDNA levels declined by 49%, and over the subsequent 4‐week period, the degradation of residual eDNA progressively slowed (Figure 3).

### 3.4 eDNA‐Based Quantification of *L. vannamei* Biomass

**FIGURE 4:**
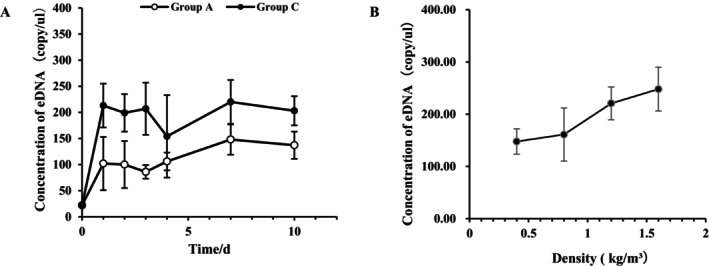
Quantitative analysis of *L.vannamei* biomass.

The aquaculture water source had a low baseline level of *L. vannamei* eDNA (22 ± 4 copies/μL), resulting in non‐zero eDNA concentrations upon the initial introduction of shrimp into the tanks. The eDNA level rose rapidly within the first day after introduction, followed by small fluctuations over the next 2–3 days, and then stabilized at a dynamic equilibrium state (Figure 4A). On Day 7 post‐introduction (Figure 4B), eDNA concentrations were measured across different stocking density treatments, enabling the establishment of a quantitative relationship between eDNA concentration and shrimp density (linear regression: y = 89.75× + 104.5, *R* ^2^ = 0.9481).

### 3.5 eDNA Release Dynamics Across Growth Stages of *L.vannamei*

**FIGURE 5:**
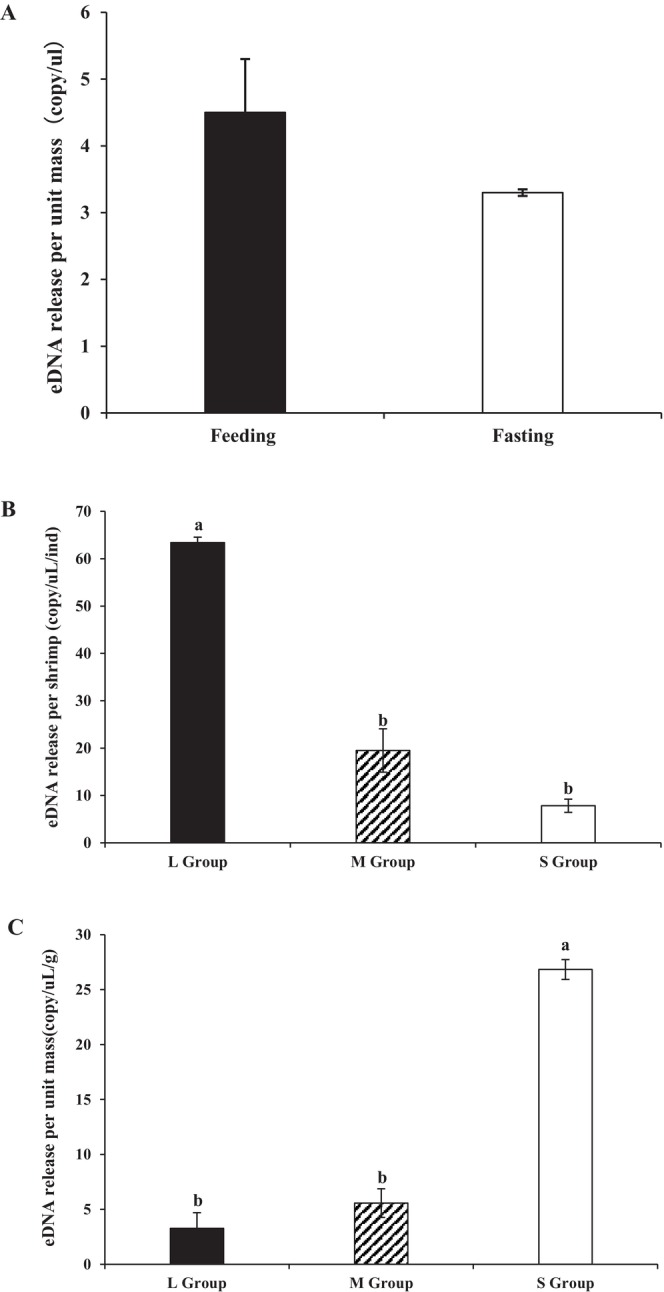
Comparative Analysis of eDNA Release Dynamics in *L.vannamei*. Group L is Large size (19.2 g/ind), Group M is Medium size (3.5 g/ind), and Group S is Small size (0.3 g/ind).

After 6 days of rearing, the eDNA release rate per unit body weight in the feeding group was higher than that in the fasting group. However, a statistical analysis (independent‐samples *t*‐test) revealed no significant difference in eDNA release per unit weight between the two groups (Figure 5A). The total eDNA release per individual of large‐sized *L.vannamei* was approximately 3.1‐fold and 8.0‐fold higher than that of medium‐sized and small‐sized individuals, respectively. In contrast, the medium‐sized group released approximately 2.5 times as much eDNA per individual as the small‐sized group (Figure 5B). With respect to eDNA release per unit body weight, small‐sized shrimp exhibited a rate approximately 7.9 times and 4.6 times higher than large‐sized and medium‐sized shrimp, respectively. Additionally, medium‐sized shrimp released eDNA at a rate approximately 1.7 times greater per unit weight compared to large‐sized shrimp (Figure 5C).

### 3.6 Temporal Dynamics of eDNA From *L.vannamei* in Indoor Aquaculture

**FIGURE 6:**
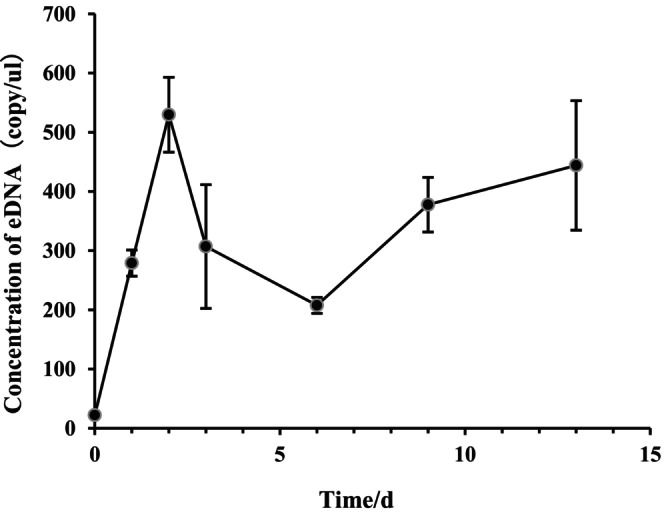
Temporal dynamics of eDNA from *L. vannamei* in outdoor factory farming systems.

Following the introduction of *L. vannamei* into the canvas pools (Figure 6), eDNA levels increased rapidly and peaked after 2 days (530 copies/μL). Over the subsequent 4 days, eDNA concentration gradually declined, reaching its minimum level on day six (208 copies/μL). Thereafter, eDNA concentration steadily increased until the conclusion of the experiment. It is worth noting that the estimated concentrations on the 3rd and 13th days have relatively high uncertainties (with wider error bars), indicating that the true values of eDNA concentrations at these two time points may fluctuate within a wider range. Therefore, caution should be exercised when interpreting the concentrations at these two time points.

### 3.7 eDNA–Biomass Relationship of *L.Vannamei* in Outdoor Earthen Ponds

The relationship between biomass yield of *L.vannamei* and eDNA concentration in outdoor earthen ponds is presented in Table 3. Pond A had a yield of 10 kg and an eDNA concentration of 234 ± 34 copies/μL at the time of sampling. Pond B yielded 7.5 kg with an eDNA concentration of 148 ± 31 copies/μL at sampling. Pond C produced no harvestable biomass and exhibited the lowest eDNA concentration (76 ± 13 copies/μL) at sampling. A positive correlation was observed between eDNA concentration and shrimp biomass, indicating that higher yields corresponded to higher eDNA levels in the pond.

**TABLE 3:** Biomass and eDNA in *L. vannamei* ponds.

Pond	eDNA concentration (copy/μL)	Output (kg)
A	234 ± 34	10
B	148 ± 31	7.5
C	76 ± 13	0

## 4 Discussion

*L. vannamei* , a cornerstone species in global aquaculture, continues to face significant challenges in achieving accurate biomass estimation under intensive farming systems (Garcia et al. 2024). Conventional monitoring approaches based on physical capture are not only operationally cumbersome but also induce physiological stress in cultured organisms. Moreover, these methods are constrained by inherent sampling variability and poor temporal resolution, limiting their ability to inform real‐time, data‐driven management decisions in modern aquaculture practices (Apún‐Molina et al. 2024; Ran et al. 2025), thereby resulting in suboptimal feeding strategies. Overfeeding, in turn, leads to the accumulation of residual feed and progressive degradation of water quality, which have emerged as key risk factors contributing to widespread disease outbreaks and substantial economic losses (Ramiro et al. 2024). Consequently, a novel, efficient, non‐invasive, and continuous biomass‐monitoring framework is urgently needed to advance sustainable shrimp aquaculture.

eDNA technology has emerged as a promising frontier tool in this context, enabling non‐invasive detection by analyzing genetic material shed by organisms into aquatic environments. It has demonstrated significant success in applications such as fish biodiversity assessment and endangered species monitoring (Dos Anjos Dos Santos et al. 2025; Sun et al. 2023). In recent years, its application in aquaculture has expanded, particularly for early pathogen detection, where it offers distinct advantages (Bohara et al. 2024). However, the direct application of eDNA technology to crustaceans—especially for the quantitative estimation of *L.vannamei* biomass—remains constrained by several technical and biological challenges. Although preliminary studies have identified a positive correlation between eDNA concentration and shrimp biomass in aquaculture systems (Liu et al. 2019), the generalizability and robustness of this relationship are influenced by multiple confounding factors. Many currently available primers are based on conserved crustacean sequences, increasing the risk of cross‐amplification and thereby compromising quantification accuracy (Komai et al. 2019). Furthermore, environmental variables such as temperature and salinity affect eDNA degradation rates, while metabolic changes across different growth stages influence eDNA shedding dynamics—both of which remain insufficiently characterized (Barnes et al. 2014; Saito and Doi 2021a). To address these limitations, this study systematically investigates the feasibility of using eDNA as a reliable tool for precise biomass assessment in *L. vannamei* aquaculture, focusing on identifying key influencing factors. The results confirm a strong correlation between eDNA concentration and shrimp biomass and further elucidate how temperature and ontogenetic stage regulate eDNA dynamics, providing a solid scientific foundation for data‐driven management in shrimp farming. A novel species‐specific primer set was designed and validated in this study, ensuring high specificity and sensitivity in detection and establishing a robust platform for downstream quantitative analyses (Figure 1). However, the primer specificity verification in this study mainly focused on a few coexisting species in water bodies. This omission may limit the direct applicability of this technique in multi‐species mixed culture systems. Furthermore, a quantitative model linking eDNA concentration to shrimp biomass was developed, revealing a significant linear positive relationship (*R* ^2^ = 0.9481) (Figure 4B), thereby demonstrating the substantial potential of eDNA technology as a non‐invasive, accurate, and scalable approach for real‐time biomass monitoring in aquaculture.

However, for the successful transition of eDNA technology from controlled laboratory settings to complex aquaculture systems, a comprehensive understanding of how environmental and biological factors influence eDNA dynamics is essential (Jo et al. 2019). This study identifies temperature as the primary environmental driver of eDNA degradation. Although eDNA degrades rapidly under elevated conditions, it remains detectable in aquatic environments for over 1 month at ambient temperature (26°C) (Figure 3), indicating a prolonged detection window. This extended persistence provides flexibility in scheduling aquaculture sampling. Nevertheless, accurate biomass estimation requires accounting for temperature‐mediated degradation rates to avoid systematic biases in quantification (Qian et al. 2022). The observed temperature‐dependent decay pattern aligns closely with variations in microbial activity and nuclease levels in water, shedding light on the natural degradation pathways and the environmental fate of eDNA in aquatic ecosystems (Saito and Doi 2021b). Furthermore, this study demonstrates that growth stage significantly influences eDNA release in *L. vannamei*, underscoring its importance for precise quantification. The eDNA release rate per unit body weight follows a clear trend: small‐sized > medium‐sized > large‐sized individuals (Figure 5C), which likely reflects intrinsic differences in metabolic activity across ontogenetic stages (Andruszkiewicz Allan et al. 2021; Korbel et al. 2024). Juvenile shrimp exhibit higher metabolic rates associated with rapid growth, leading to more frequent cellular turnover, excretion, and mucus production per unit mass—processes that contribute to elevated eDNA shedding (Barnes and Turner 2016). In contrast, larger shrimp display relatively lower metabolic activity, resulting in reduced eDNA release per unit biomass (Takahara et al. 2012).

In actual production, the growth rate and physiological state of shrimp may vary significantly due to environmental temperature and other conditions (such as salinity, ammonia nitrogen, etc.), which further affects the release characteristics of eDNA. Recent studies have pointed out that high‐temperature stress can significantly alter the physiological homeostasis of shrimp, such as by disrupting the integrity of the intestinal mucosa, regulating immune signaling pathways, and changing the composition of the intestinal microbiota, thereby influencing overall metabolic activities and the excretion of metabolic products (including eDNA) (Duan et al. 2025). Additionally, stress factors in the environment, such as salinity and ammonia nitrogen, can also regulate the energy metabolism and detoxification metabolism of shrimp, thereby indirectly altering the production rate of eDNA (Andruszkiewicz Allan et al. 2021). Therefore, in actual production environments, different temperature gradients and water quality conditions may lead to differences in eDNA release among shrimp of the same weight. This additional variability should be considered as a potential confounding factor to further improve the quantitative model of eDNA. Therefore, accurate biomass assessment using eDNA requires the development of growth‐stage‐specific calibration models. Notably, this study further reveals that short‐term feeding status does not significantly affect eDNA release per unit mass (Figure 5A), consistent with previous findings that acute feeding events are not primary determinants of eDNA output (Mächler et al. 2018). This finding simplifies practical implementation by reducing the need for strict feeding controls, enabling reliable sampling in operational aquaculture ponds without fasting periods. The result shown in Figure 5C, that “the smaller the body size, the more eDNA released per unit weight”, is not simply related to metabolic activities. This phenomenon is actually the result of the combined effects of multiple biological factors. For crustaceans, molting is a drastic physiological process. Small shrimp grow rapidly and molt frequently. The molting process not only involves the shedding of the old shell but also the intense remodeling of the epidermal cells and extensive apoptosis, which significantly enhances their eDNA release efficiency (Stevenson 1972). The surface area to volume (mass) ratio (S/W) is also a key physical principle that determines the physiological requirements of organisms. As the size of an individual increases, the volume (mass) grows at a much faster rate than the surface area, resulting in a significant decrease in the S/W ratio. This means that small shrimp must conduct gas exchange and waste excretion through a relatively larger surface area (Emerson 1967). At the same time, the sex ratio and behavioral activity cannot be ignored. In large shrimp groups, the proportion of males may be higher, resulting in a greater overall activity level but diluted by a larger body weight, while medium‐sized shrimp may contain more females, and the metabolic characteristics of females during egg storage or spawning are different from those of males (Chen et al. 2026). This coupling effect of body size and sex further explains why the release rate of medium‐sized shrimp is approximately 1.7 times that of large shrimp.

In summary, this study integrates multidimensional evidence—including primer specificity validation for *L. vannamei*, the effects of environmental variables, biological sources of eDNA release, and both laboratory‐scale and field‐based validation—to establish a comprehensive framework for eDNA‐based biomass assessment. The consistent linear relationship observed in controlled mesocosm experiments, together with the positive correlation between eDNA concentration and production in outdoor aquaculture ponds (Table 3), not only confirms the approach's technical reliability but also highlights its practical applicability in real‐world shrimp farming systems.

## 5 Conclusion

This study employed *L. vannamei* as a model species. It used qPCR to evaluate the feasibility and performance of an eDNA‐based approach for quantifying shrimp biomass. Results showed that after removing the eDNA source, *L. vannamei* eDNA concentrations in water declined gradually, with detectable levels persisting for over 1 month. Furthermore, experiments across three temperature gradients revealed that higher temperatures accelerated eDNA degradation, indicating a positive correlation between temperature and decay rate. A significant positive relationship was observed between shrimp biomass and aqueous eDNA concentration. Notably, eDNA release rates varied among size classes: the large‐sized group exhibited a per‐unit‐body‐weight release rate approximately 7.9 times that of the medium‐sized group and 4.6 times that of the small‐sized group. No significant difference in eDNA release was detected between feeding and fasting groups after 6 days of cultivation. In practical applications, eDNA technology offers substantial advantages over traditional trawl‐based sampling, including reduced time and labour requirements, greater cost‐effectiveness, operational simplicity, and higher sensitivity. It is therefore suitable for qualitative biomass assessment in *L. vannamei* aquaculture. However, due to limitations such as a relatively small sample size and potential confounding factors—including unscheduled shrimp mortality caused by environmental fluctuations or management practices—the accuracy of quantitative biomass estimation requires further refinement through additional research.

## Author Contributions

**Guangyan Liang:** data curation (equal), writing – original draft (equal), writing – review and editing (equal). **Sizhe Wang:** investigation (equal), methodology (equal), validation (equal). **Shan Wang:** formal analysis (equal), supervision (equal), writing – review and editing (equal). **Yuxue Qin:** supervision (equal), validation (equal), writing – review and editing (equal). **Yusheng Jiang:** funding acquisition (equal), project administration (equal), writing – review and editing (equal).

## Funding

This work was supported by the National Natural Science Foundation of China, 42276145. The Major Special Project of Science and Technology of Liaoning Province—Germplasm Innovation Project, 2024JH1/11700009. The National Key Research and Development Program of China (Intergovernmental Cooperation on International Science and Technology Innovation), Ministry of Science and Technology of China (2022–2025), for the project “Development of a high‐density intelligent recirculating aquaculture system using high dissolved oxygen equipment and IoT‐based water quality management”, 2022YFE0117900.

## Ethics Statement

The authors have nothing to report.

## Conflicts of Interest

The authors declare no conflicts of interest.

## Supporting information


**Data S1:** ece373299‐sup‐0001‐DataS1.xlsx.

## Data Availability

All the required data are uploaded as Supporting Information.
